# In Vivo Efficacy of Neutrophil-Mediated Bone Regeneration Using a Rabbit Calvarial Defect Model

**DOI:** 10.3390/ijms222313016

**Published:** 2021-12-01

**Authors:** Thanuja D. K. Herath, Leonardo Saigo, Benoit Schaller, Anis Larbi, Swee Hin Teoh, Charles James Kirkpatrick, Bee Tin Goh

**Affiliations:** 1 National Dental Centre Singapore, 5 Second Hospital Avenue, Singapore 168939, Singapore; thanuja.herath@ndcs.com.sg (T.D.K.H.); Leonardo.Saigo@singhealth.com (L.S.); 2 Department of Cranio-Maxillofacial Surgery, Inselspital, Bern University Hospital, University of Bern, 3010 Bern, Switzerland; Schaller.Schaller@insel.ch; 3 Singapore Immunology Network, Agency for Science Technology and Research, Singapore 138632, Singapore; anislarbi69@gmail.com; 4 School of Chemical and Biomedical Engineering, Nanyang Technological University, Singapore 637459, Singapore; teohsh@ntu.edu.sg; 5 Lee Kong Chian School of Medicine, Nanyang Technological University, Singapore 636921, Singapore; 6 Clinic for Cranio-Maxillofacial Surgery, University Medical Centre, Johann Wolfgang Goethe University, 60323 Frankfurt, Germany; kirkpatrick@uni-mainz.de

**Keywords:** neutrophils, bone regeneration, osteogenesis, rabbit calvarial defect model

## Abstract

Reconstruction of bone due to surgical removal or disease-related bony defects is a clinical challenge. It is known that the immune system exerts positive immunomodulatory effects on tissue repair and regeneration. In this study, we evaluated the in vivo efficacy of autologous neutrophils on bone regeneration using a rabbit calvarial defect model. **Methods:** Twelve rabbits, each with two surgically created calvarial bone defects (10 mm diameter), were randomly divided into two groups; (i) single application of neutrophils (SA-NP) vs. SA-NP control, and (ii) repetitive application of neutrophils (RA-NP) vs. RA-NP control. The animals were euthanized at 4 and 8 weeks post-operatively and the treatment outcomes were evaluated by micro-computed tomography, histology, and histomorphometric analyses. **Results:** The micro-CT analysis showed a significantly higher bone volume fraction (bone volume/total volume) in the neutrophil-treated groups, i.e., median interquartile range (IQR) SA-NP (18) and RA-NP (24), compared with the untreated controls, i.e., SA-NP (7) and RA-NP (14) at 4 weeks (*p* < 0.05). Similarly, new bone area fraction (bone area/total area) was significantly higher in neutrophil-treated groups at 4 weeks (*p* < 0.05). Both SA-NP and RA-NP had a considerably higher bone volume and bone area at 8 weeks, although the difference was not statistically significant. In addition, immunohistochemical analysis at 8 weeks revealed a higher expression of osteocalcin in both SA-NP and RA-NP groups. **Conclusions:** The present study provides first hand evidence that autologous neutrophils may have a positive effect on promoting new bone formation. Future studies should be performed with a larger sample size in non-human primate models. If proven feasible, this new promising strategy could bring clinical benefits for bone defects to the field of oral and maxillofacial surgery.

## 1. Introduction

Reconstruction of large bone defects in the oral and maxillofacial region as a result of trauma, bone tumors, or congenital deformities remains a major clinical challenge [1]. Although autologous bone or synthetic bone grafts have yielded satisfactory results for bone regeneration, they often possess significant limitations in terms of availability, efficacy, immunological reactions, and risk of disease transmission [2]. Moreover, additional surgery for bone harvesting also causes pain and donor site morbidity. For decades, researchers have sought suitable alternatives. Bone tissue engineering has been considered a promising strategy to overcome foregoing challenges in the reconstruction of bone defects.

Hitherto, various approaches in bone tissue engineering have been evaluated, including cell- and growth factor-based strategies [3,4,5]. Recently, it has been proposed that 3D co-culture systems containing multiple cells are a promising strategy [6,7]. Cell-based approaches in bone tissue engineering primarily target the early stages of bone repair, which initiates the inflammatory driven bone regenerative cascade. Until now, several studies have investigated the ability of different cell types, i.e., embryonic stem cells, induced pluripotent stem cells, mesenchymal stem cells, and endothelial progenitor cells, in order to enhance bone repair and regeneration. Despite the advantages of cell-based approaches, there are unresolved issues such as limitations in the isolation and expansion of cells, the expression and stability of osteogenic markers, and long-term safety in terms of immune rejection, graft-versus-host disease, and tumorigenicity [8,9]. Although some encouraging results have been obtained in vitro, the in vivo efficacy of the aforementioned techniques is limited [10].

The biology of bone healing is a complex process that follows specific regenerative patterns and includes an initial inflammatory phase, and repair and remodeling of the tissues [11]. A neutrophil-predominant, inflammatory reaction is the first healing response after an injury. Subsequently, a hematoma is formed, and neutrophils, among other cell types, infiltrate into the tissue and trigger an inflammation-associated bone repair and regenerative cascade at the site of injury [12,13,14]. Infiltrated neutrophils result in an elevated expression of signaling molecules involved in the regulation of bone formation (i.e., pro-inflammatory cytokines including interleukins (ILs), transforming growth factor beta (TGF-β), tumor necrosis factor alpha (TNF-α), chemotactic chemokines, fibroblast growth factor (FGF-2), and platelet-derived growth factor (PDGF)) [13]. This micro-environment initiates new tissue formation and remodeling.

Recently, it was found that neutrophils are constituted of bona fide subsets that possess pro-angiogenic properties [15,16]. This specific population of angiogenic neutrophils secretes matrix metalloproteinase-9 (MMP-9) into the extracellular matrix (ECM) to liberate pro-angiogenic growth factors, vascular endothelial growth factor (VEGF) and fibroblast growth factor-2 (FGF-2) into the extracellular matrix, which promote angiogenesis [17,18,19]. Although the foregoing studies have suggested the key role played by neutrophils in angiogenesis and the bone healing processes, conclusive evidence has yet to be demonstrated. Previously, using a triple-cell co-culture model consisting of osteoblasts, endothelial cells, and neutrophils, we demonstrated the promising osteogenic and angiogenic potential of neutrophils in vitro [20]. Thus, neutrophils significantly increased angiogenesis and osteogenesis in the tissue construct, evidenced by the formation of microvessel-like structures and a mineralized matrix deposition. Considering the foregoing information and our encouraging in vitro data, in this pilot study, we sought to evaluate the in vivo efficacy of neutrophil-mediated bone tissue regeneration.

## 2. Results

### 2.1. Clinical Observation

All animals recovered from anesthesia and the surgical procedure, and remained healthy and active during the observation period. There were no signs of infection or wound dehiscence during the wound healing process in any of the animals.

### 2.2. Micro-CT

**Figure 1:**
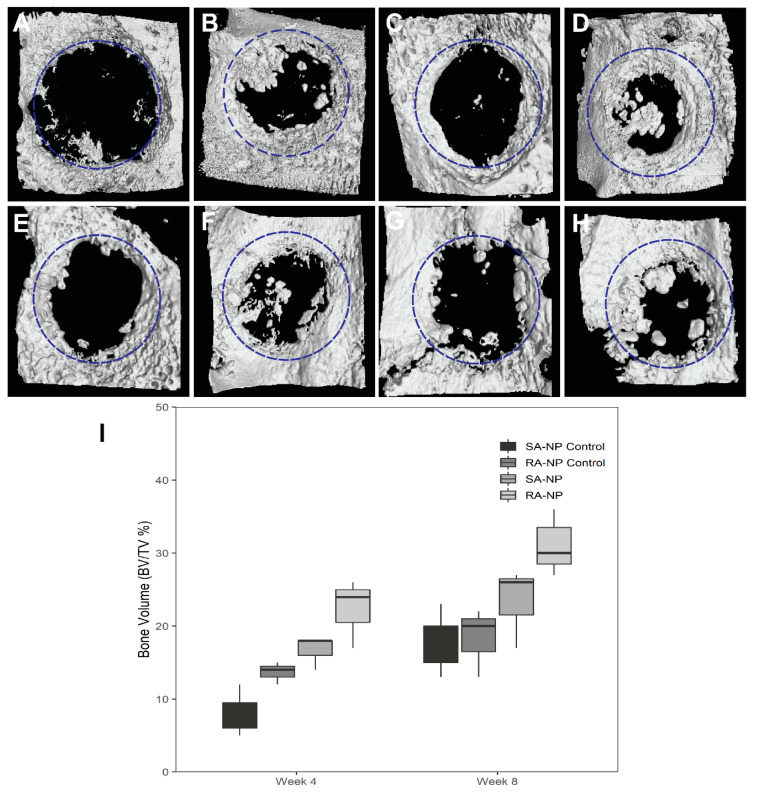
Representative micro-CT images of calvarial defects at 4 weeks (**A**–**D**) and 8 weeks (**E**–**H**) showing: the center of the defect of the (**A**,**E**) SA-NP control, (**B**,**F**) single application of neutrophils (SA-NP), (**C**,**G**) RA-NP control, (**D**,**H**), and repetitive application of neutrophils (RA-NP). (**I**) New bone volume measured by micro-CT for different experimental groups. The volume of new bone was expressed as a percentage of the total volume within the defect. Statistically significant difference in percentage of new bone volume fraction (BV/TV, %) in neutrophil treated groups compared with respective controls at both 4 and 8 weeks after healing (*p* < 0.05).

Micro-CT images of the calvarial defect at 4 and 8 weeks postoperatively are shown in Figure 1. The circles superimposed on each of the representative micro-CT images indicate the margin of the original bone defect. Newly generated bone was formed only at the margins of the defect in the control groups, whereas the SA-NP and RA-NP groups showed signs of bony repair both at the periphery and the center of the defect (Figure 1B,D,F,H). Interestingly, neutrophil-treated groups had a significantly higher bone volume fraction at 4 weeks compared with the controls (*p* < 0.05). There was significantly higher bone volume fraction (BV/TV) in the neutrophil-treated groups (Figure 1I). The median interquartile range (IQR) was used to analyze the data, which showed the higher BV/TV of SA-NP (18) and RA-NP (24) groups compared to the untreated SA-NP control (7) and RA-NP control (14) at 4 weeks (*p* < 0.05) (Supplementary Table S1). The bone volume fraction was higher in the RA-NP group compared with the SA-NP group at 4 weeks, but the difference was not statistically significant (Figure 1D). After 8 weeks postoperatively, control groups (Figure 1E,G) showed a slight increase in the newly formed bone. In contrast, neutrophil-treated groups showed a considerably high bone formation (Figure 1F,H). The IQR of the SA-NP and RA-NP groups were higher than the SA-NP control and RA-NP control groups at 8 weeks (Figure 1I). Although the difference was not statistically significant, there was a clear gain in the bone volume of the neutrophil-treated groups (*p* > 0.05).

### 2.3. Descriptive Histology

**Figure 2:**
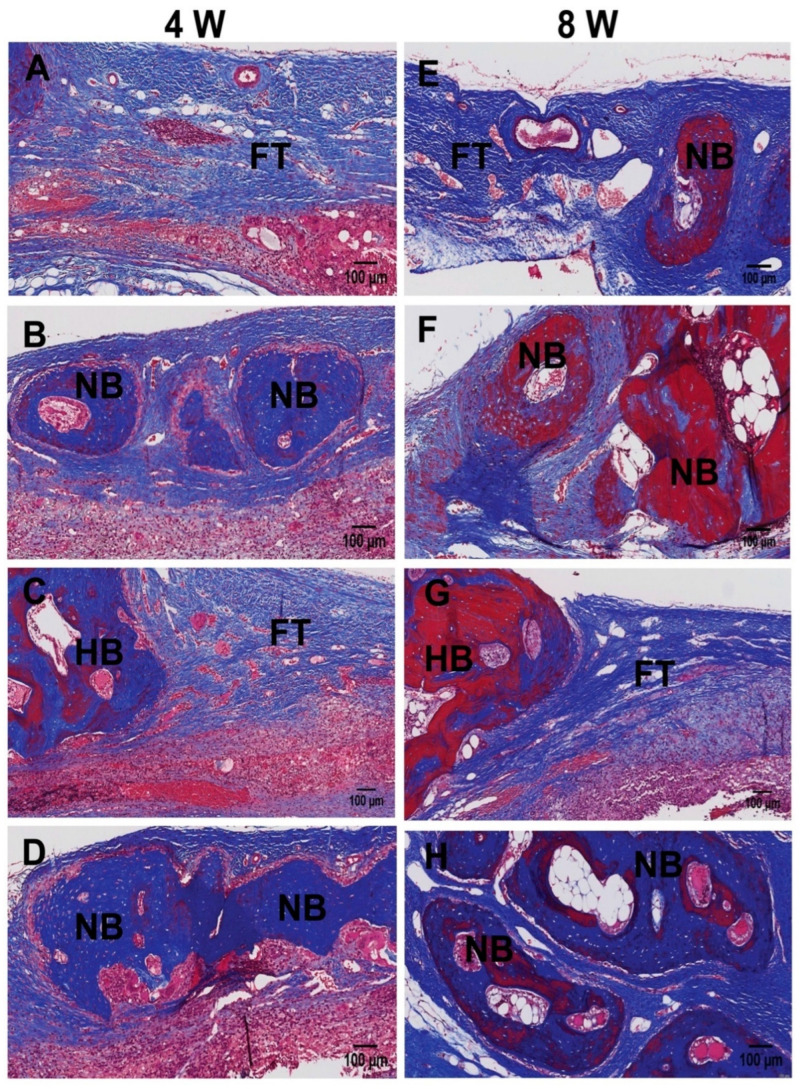
Histological sections (Masson’s Trichrome staining) of rabbit calvarial defect and the surrounding tissue at 4 weeks (**A**–**D**) and 8 weeks (**E**–**H**). (**A**,**E**) SA-NP control, (**B**,**F**) single application of neutrophils (SA-NP), (**C**,**G**) RA-NP control, (**D**,**H**), and repetitive application of neutrophils (RA-NP). NB, new bone; HB, host bone; FT, fibrous tissue (scale bar 100 μm).

**Figure 3:**
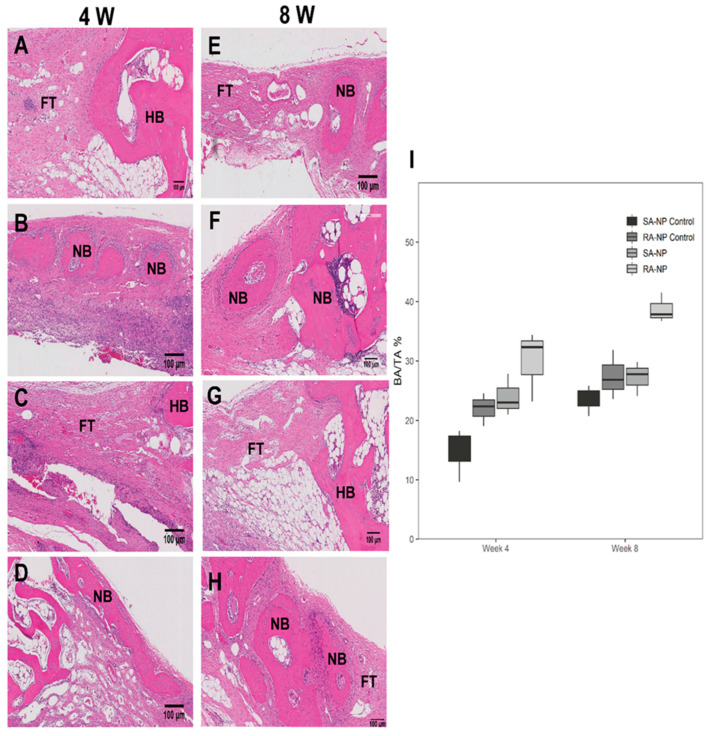
Histological sections (H&E staining) of rabbit calvarial defect and the surrounding tissue at 4 weeks (**A**–**D**) and 8 weeks (**E**–**H**). (**A**,**E**) SA-NP control, (**B**,**F**) single application of neutrophils (SA-NP), (**C**,**G**) RA-NP control, (**D**,**H**) and repetitive application of neutrophils (RA-NP). NB, new bone; HB, host bone; FT, fibrous tissue (scale bar 100 μm). (**I**) Histomorphometric analysis of the bone area fractions in different experimental groups. The area of new bone was expressed as a percentage of the total area available for tissue ingrowth within the defect (BA/TA, %). Statistically significant difference in the percentage of new bone area fraction in the neutrophil treated groups compared with the controls at both 4 and 8 weeks after healing (*p* < 0.05).

Representative histological images of Masson’s Trichrome and H&E staining are shown in Figure 2 and Figure 3. The defect sites in the control were observed to be filled mostly with fibrous connective tissues rather than the newly formed bone at 4 weeks (Figure 2A,C and Figure 3A,C). Histological analysis revealed minimal amounts of bony ingrowth and very few bony islands at the defect side (Figure 2A,C and Figure 3A,C). In contrast, neutrophil-treated groups showed considerable new bone formation at both 4 (Figure 2B,D and Figure 3B,D) and 8 weeks (Figure 2F,H and Figure 3F,H). At 4 weeks, circular new bony islands with the presence of early woven bone was observed, originating at the defect margins. At 8 weeks of healing, newly formed lamellar bone was observed (Figure 2F,H and Figure 3F,H). The RA-NP group showed more new bony islands compared with the SA-NP group (Figure 2D,H and Figure 3D,H). Bone maturation was more extensive at 8 weeks of healing.

### 2.4. Histomorphometry

Histomorphometric analysis of the bone area fractions of the defects are presented in the Box-plot analysis (Figure 3I and Supplementary Table S1). There were higher bone area fractions in the neutrophil-treated groups compared with the respective controls (Figure 3I). The difference between the neutrophil-treated groups and untreated controls was significant at 4 weeks (*p* < 0.05). Although there was a considerable difference between the neutrophil-treated groups and controls at 8 weeks, the difference was not statistically significant (*p* > 0.05).

### 2.5. Local Expression of OCN

**Figure 4:**
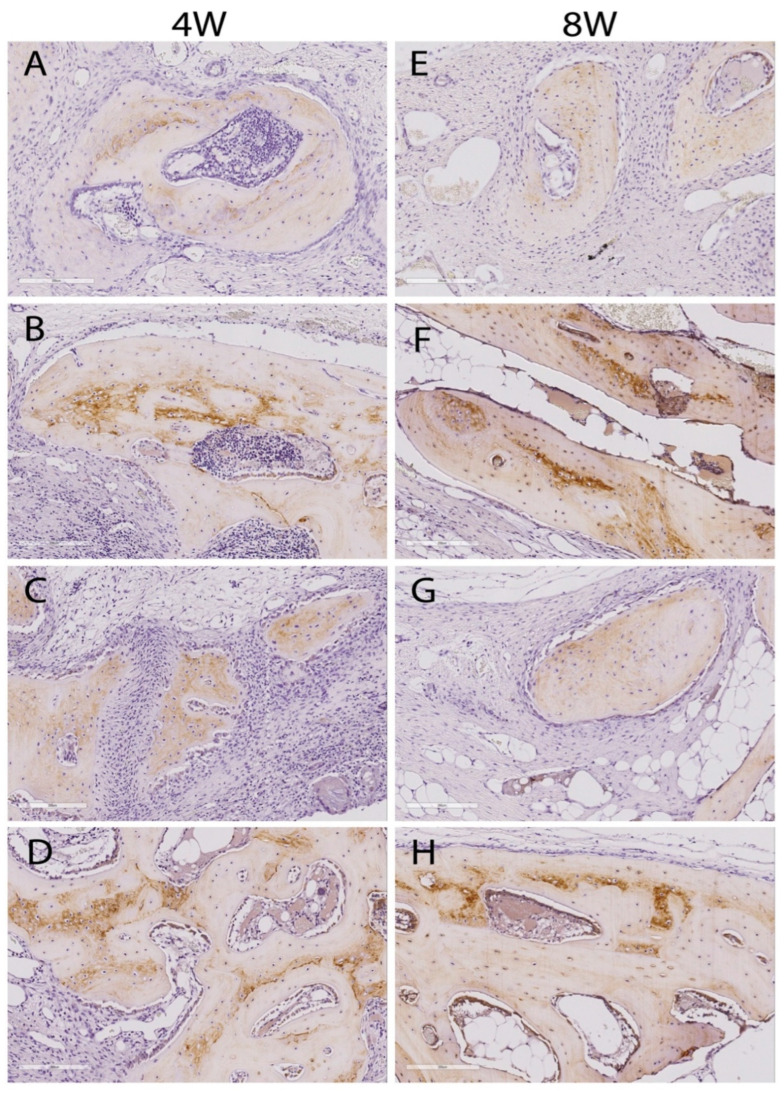
Immunohistochemical staining of osteocalcin (OCN) at 4- and 8-weeks post-surgery. Brown color represents the positive staining for the OCN protein. OCN is highly expressed in the osteoblasts at the margin of the newly formed bony islands in the SA-NP (**B**,**F**) and RA-NP (**D**,**H**) groups, whereas limited positive staining is seen in the SA-NP (**A**,**E**) and RA-NP (**C**,**G**) control defect sites at both 4 and 8 weeks (scale bar 100 μm).

New bone formation in the calvarial defect was also analyzed by immunohistochemical staining for OCN expression (Figure 4). OCN is a key marker for the mature osteoblast phenotype, specifically in the lamellar bone. Therefore, OCN staining can help to detect osteogenesis in the regenerated bone. The appearance of a yellow-brown substance indicates positive staining. In the control groups, minimal OCN expression was found (Figure 4A,C,E,G). The SA-NP group showed increased levels of OCN expression in osteoblasts at both 4 and 8 weeks (Figure 4B,F). Compared with the control groups and the SA-NP group, the RA-NP group showed a stronger OCN expression (Figure 4D,H).

## 3. Discussion

Ready-to-use cell-based implantation therapies are an urgent requirement in the field of bone tissue engineering. In the present study, we examined the in vivo efficacy of autologous neutrophils on bone repair and regeneration using a rabbit calvarial defect model. The critical-sized 10 mm calvarial bone defect is a well-established model for the study of bone augmentation that will not heal spontaneously, regardless of the time given for healing. It was obvious from the findings that healing of the control defects was considerably less than for the neutrophil-treated groups. Hence, the results demonstrated that the neutrophils have a positive effect on in vivo bone regeneration. There was a statistically significant higher bone regeneration in the neutrophil-treated groups, SA-NP and RA-NP compared with the untreated controls at 4 weeks. Although a considerable bone formation was seen in the neutrophil-treated rabbit calvarial defects at 8 weeks, the difference did not reach statistical significance, probably due to the small sample size in the study.

The comparative differences in the bone regeneration between neutrophil-treated and untreated groups were observed in the micro-CT analysis as well as histomorphometric analysis. In the untreated control groups, there was minimal bone regeneration, with no obvious reduction of the defect area. The new bone volume fraction (BV/TV) as well as new bone area fraction (BA/TA) in the neutrophil-treated groups were found to be significantly larger than for the untreated control groups at 4 weeks (*p* < 0.05). Histologically, Masson’s Trichrome staining for bone showed large amounts of bony nodules in the neutrophil-treated groups. As the primary aim of the present study, more focus was placed on examining these bony islands. The newly formed bone tissue exhibited morphological features of the normal bone. Dense connective tissues could be seen in the surrounding area. Bone tissue contained osteoblasts lining the outer edge and osteocytes embedded in their lacunae. The presence of mature osteoblast phenotypes was indicated by positive staining for OCN, particularly in the newly formed bone in the neutrophil-treated groups.

Interestingly, repetitive application of neutrophils (RA-NP group) regenerated a higher percentage of bone within the defect compared with the controls and single application of neutrophils, i.e., the SA-NP group. This observation can be explained based on the functional role and the contribution of neutrophils during the early stages of the bone healing process. The biology of bone healing follows a specific regenerative pattern, and a neutrophil-predominant, inflammatory response is the first healing response after an injury [11]. In a normal situation where there is tissue damage, the hematoma releases cytokines to recruit neutrophils to the wound bed in order to remove possible invasion of microorganisms. This is indicative of the early inflammatory responses. Even though neutrophils have a short life span (7–12-h half-life) upon migration into tissues, it has been shown that neutrophils could remain active in the wound site for considerably lengthy periods of time, up to 2–3 days [21]. This is evidenced by continuous recruitment of neutrophils to the site, as well as inhibition of the normal spontaneous apoptosis of neutrophils during the resolution of inflammation [22,23]. Therefore, supplementation of an additional neutrophil population is an advantage in order to continue the pro-inflammatory response at the core of the defect in the early period following injury. This pro-inflammatory response is known to trigger bone healing and the regenerative cascade. Neutrophils are able to secrete a myriad of pro-inflammatory mediators, pro-angiogenic growth factors, and osteogenic factors which initiate and trigger the bone regenerative cascade [12,13]. Hence, the data derived from the present study indicate that the addition of supplementary doses of neutrophils at early stages of bone healing may provide a beneficial effect on immune-modulated bone regeneration.

The in vivo results of the present study are in line with our previous in vitro data that demonstrated the positive effect of neutrophils on osteogenesis and angiogenesis in a triple cell co-culture model [20]. Kovtun et al. (2017) also demonstrated that neutrophil reduction considerably impaired bone regeneration in a rat femur defect model [24]. One of the most important aspects in the healing process is the fracture hematoma formed during the induction stage [25]. Kolar et al. (2010) found that washing out the fracture hematoma delayed the early healing process of fractures [14]. The hematoma acts as a temporary scaffold for the active invasion of additional inflammatory cells. The first cells recruited are polymorphonuclear neutrophils (PMNs), which are attracted by a cytokine/chemokine gradient response and rapidly accumulate during the first hours after injury. Although PMNs are short-lived cells, they secrete several cytokines and chemokines, including IL-1, TNF-α, IL-8, CCL2, and IL-6, and growth factors such as VEGF-A and MCP1 to initiate and direct the bone repair and regeneration cascade [15,17]. Therefore, modulation in the early inflammatory phase of bone repair may influence downstream processes of fracture healing.

In the present study, we used neutrophils in the fibrin gel as a delivery system. It has been shown that seeding cells in an encapsulated material such as fibrin gel is an efficient and successful approach in tissue engineering [26,27,28]. As previously shown by He et al., the fibrin carrier increases the surface area for cell attachment and provides growth factors to promote cell differentiation and bone regeneration [29]. Hence, fibrin gel has been used in similar studies in tissue engineering, as it protects the cells from external forces, facilitates uniform cell distribution, and readily degrades during wound healing with minimum inflammation [27,30]. Thus, we took advantage of this strategy to mix the neutrophils with fibrin gel for delivery into the calvarial defect site. Some studies reported the ability of fibrin gel to promote new bone formation. However, we still have to examine if fibrin gel itself promotes osteoblast differentiation and accelerates new bone formation.

Although the neutrophil-treated groups showed considerable new bone formation in the bone defects, the amount of new bone formed was not adequate to completely heal the defect. This may be due to the relatively short incubation period of 8 weeks, which is a limitation of the study. Therefore, future studies are warranted so as to examine the long-term efficacy of neutrophil-mediated bone regeneration approaches. A small sample size is another limitation of this study, and future studies with a larger sample size are warranted.

To our knowledge, this is the first study to evaluate the in vivo efficacy of neutrophils on bone regeneration using a rabbit calvarial defect model. This study has given new insight into the possible immune-modulated bone regenerative strategies. The application of autologous neutrophils for bone defect healing and regeneration has many advantages over autografts, allografts, xenografts, and synthetic bone grafts. This strategy circumvents the additional surgery needed to collect the bone graft and reduces the risk of disease transmission and immune rejection.

## 4. Materials and Methods

### 4.1. Management of Animals

Twelve male New Zealand white rabbits (*Orytolagus cuniculus*) weighing between 3.0–3.5 kg were used in this study. According to the guidelines, the rabbits were kept in separate cages under standard laboratory conditions and were fed a standard rabbit diet throughout the duration of this study. The selection of experimental animals, their management, and the surgical protocol was approved by the National Advisory Committee on Laboratory Animal Research (NACLAR) Singapore.

The animals were randomly divided into two groups of six animals each viz. (i) single application of neutrophil group (SA-NP) (*n* = 3), where a single dose of neutrophils mixed with fibrin gel was added to the defect at once, while the contralateral defect was filled with phosphate-buffered saline (PBS) with fibrin gel as the control (SA-NP control) (*n*), and (ii) repetitive application of neutrophil group (RA-NP) (*n* = 3), where a single dose of neutrophils was added to the defect three times on day 0, 2, and 4. Day 0 was similar to SA NP group, where neutrophils mixed with fibrin gel was added to the defect, while the contralateral defect was filled with phosphate-buffered saline (PBS) with fibrin gel as the control. At days 2 and 4, a single dose of neutrophils mixed with PBS was injected to the defect side, whereas the contralateral side was treated with PBS on the same days (RA-NP control) (*n* = 3).

### 4.2. Surgical Procedure

**Figure 5:**
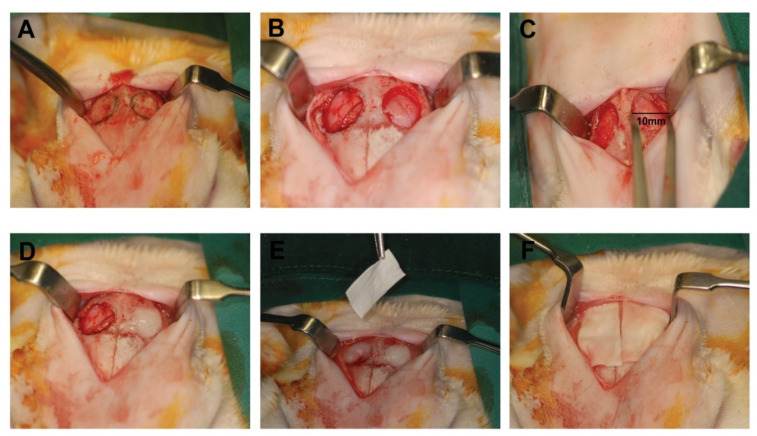
Surgical procedure. (**A**–**C**) The appearance of the bilateral 10-mm critical-sized calvarial defects in rabbit cranium. (**D**) One side of the defect was filled with either SA-NP or RA-NP with fibrin gel, and the contralateral side was filled with fibrin gel alone. (**E**,**F**) Application of collagen membrane onto the defect.

Two bilateral circular calvarial defects were made in the parietal bone on each side of the median sagittal suture without crossing it (Figure 5). In brief, animals were fasted overnight and received preoperatively 0.2 mg/kg of intramuscular medetomidine hydrochloride (Domitor^®^) (Orion-Farmos, Espoo, Finland) and 10 mg/kg of intramuscular ketamine pre-operatively. An oral endotracheal tube was used to perform endotracheal intubation and 1–3% isoflurane was used to maintain anesthesia. The animals were given 2 mg/kg of tramadol and 5 mg/kg of enrofloxacin (Baytril^®^) (Bayer HealthCare LLC, Emeryville, CA, USA). The skull regions of the animals were shaved and povidone-iodine and 0.05% chlorhexidine gluconate solutions were used to clean the area. A midline incision was performed in the skin of the calvarium along the sagittal suture line. In order to ensure a uniform defect size, a pre-fabricated surgical guide was employed. Using a surgical burr, circular osseous bony defects of 10 mm in diameter were made using a surgical burr on each side of the sagittal suture by standard methodology so as to avoid overheating and to preserve dura.

### 4.3. Isolation of Autologous Neutrophils from Rabbit Whole Blood

Autologous neutrophils were isolated from the animals by standard protocol, as previously described [31,32]. Blood collection from the rabbits and isolation of neutrophils were performed on the same day as the cranial defect surgery in order to ensure the viability and to minimize the activation of neutrophils. In brief, 15 mL of peripheral blood was drawn from the ear artery of each animal by venipuncture [33]. Blood was then transferred into heparin/EDTA anticoagulant treated blood collection tubes and Optiprep^TM^ (Axis-Shield, Dundee, Scotland) was used to separate the neutrophils from the peripheral blood mononuclear cells by density gradient centrifugation, according to the optimized protocol from our group [20,34]. In brief, blood was layered on dextran and incubated for 30 min to sediment the erythrocytes at first. Subsequently, the leukocyte-rich plasma was layered on Optiprep^TM^ and then centrifuged for 30 min at 500× *g*, at 22 °C. The Hanks balanced salt solution without Ca^2+^/Mg^2+^ was used to re-suspend the neutrophils. Residual erythrocytes were removed with a red blood cell lysis buffer and the cell concentration was estimated using a hemocytometer. According to the standard methodology, 0.1% trypan blue dye staining was used to examine the viability of the isolated neutrophils.

### 4.4. Application of Neutrophils to the Bony Defects

A total of 12 million neutrophils (12 × 10^6^ cells) were mixed with 0.1 mL PBS to prepare the standardized neutrophil suspension. For the repetitive step, we used freshly isolated neutrophils from the same animal to obtain an autologous neutrophil sample, similar to the procedure followed on day 0. Hence, for the repetitive administration of neutrophils (RA-NP), a fresh blood sample was collected each day at days 2 and 4 to prepare the fresh neutrophil suspension. Fibrin gel was prepared using TISSEEL kit according to the manufacturer’s instructions (Baxter International Inc., Deerfield, IL, USA). Equal volumes (0.05 mL) of human fibrinogen and thrombin solutions were first mixed together in a mini petri dish to form a semi-solid fibrin gel (0.1 mL) as follows:

(i) SA-NP group: The standardized neutrophil suspension (0.1 mL) was mixed with a semi-solid fibrin solution and was allowed to form a gel for 3–4 min. Afterwards, the completely settled fibrin gel/neutrophil mixture was applied into the calvarial bony defect. The control group was given 0.1 mL PBS mixed with fibrin gel. Each defect was covered with a collagen membrane (Geistlich Bio-Gide^®^, Geistlich Pharma AG, Division Biomaterials, Wohusen, Switzerland). The collagen membranes came with a standard dimension of 25 mm × 25 mm. The membrane was appropriately cut to fit the calvarial defects.

(ii) RA-NP group: A standardized dose of 12 × 10^6^ neutrophils in PBS (0.1 mL) fibrin gel was added to the defect side at day 0, whereas the contralateral side was treated with PBS (0.1 mL) alone. At days 2 and 4, a standardized dose of 12 × 10^6^ neutrophils in PBS without fibrin gel was added into the defect side using the standard injection method. We targeted mainly the center of the defect for efficient release and retention of neutrophils.

### 4.5. Harvesting Tissues for Downstream Analysis

The animals were carefully monitored throughout the postoperative healing period for any signs of adverse complications such as allergic reactions, and other complications. The eating habits and stress levels were monitored using rabbit pain assessment (rabbit grimace scale). The animals received antibiotics (Baytril, 5 mg/kg, IM) for 6 days post-operatively, and analgesics (Tramadol, 1–3 mg/kg, IM) for 2–5 days post-operatively. Three animals from each group were sacrificed at two time points, viz. 4 weeks and 8 weeks. The animals were sacrificed according to a standard protocol. Harvested tissue sections were fixed with 10% neutral buffered formalin solution and wereprocessed for subsequent micro-computed tomography (micro-CT), histological, histomorphometric, and immunohistochemical analyses.

### 4.6. Evaluation Methods

#### 4.6.1. Micro-CT Scanning

Micro-CT images were obtained for the entire specimen using a nanoscan SPECT/CT system (Mediso Medical Imaging Systems, Budapest, Hungary). The specimens were secured to the sample holder and attached to the micro-CT animal bed. A semi-circular scan with 720 X-ray projections at a maximum field of view of 63 mm was used with a 50 kV current and 300 ms exposure time. The binning factor was set to 1:1 for a higher resolution. Reconstruction of the CT slices was performed immediately in the system with the set parameters of medium in-plane voxel size and medium slice thickness to obtain CT data with 40 µm spatial resolution. After acquisition and reconstruction, the data were exported for processing and analysis for bone volume fraction (BV/TV) in the defective region.

The images were produced in 0.4 mm slices and were reconstructed in a computer using VG studio 2.2 3D Image Viewer and Analysis Tool version 2.2 (GE Healthcare, Waukesha, WI, USA). A customized region of interest (ROI) was drawn at the defect site. The ROI was cylindrical in shape, measuring 10 mm in diameter with 2 mm thickness to cover the whole defect area. BV/TV was determined using the software. This represented the percentage of mineralized bone detected at the defect site. BV/TV was calculated by measuring the bone volume (BV) divided by the total volume (TV) in the selected region of interest.

#### 4.6.2. Histological Analysis

After micro-CT scanning, the specimens were decalcified by a slow decalcification reagent using 10% EDTA for 28 days. It was then dehydrated using an ascending series of alcohol, cleared in xylene, and embedded in paraffin. Leica RM2255© microtome (Leica Microsystems GmbH, Wetzlar, Germany) was used for microtomy of the specimens. The specimens were cut from the center of each defect and serial sectioning, starting from the central part to a final thickness of 5 µm. Each section contained three different slices at intervals of 20 µm to obtain maximum standardization of the cutting surface. Sections were stained with hematoxylin and eosin (H & E) and Masson’s Trichome for descriptive histology. Masson’s Trichome stained specimens were used to analyze the final bone area fraction (BA/TA), which was calculated by measuring the bone area (BA) divided by the total area (TA) in the selected region of interest.

Representative slides were prepared for each group and the final bone area fraction was determined.

#### 4.6.3. Histomorphometric Analsysis

The Masson’s Trichome slides were used for the histomorphometrical analysis. Slides were scanned at 20× using a Leica SCN400 slide scanner (Leica Microsystems GmbH, Wetzlar, Germany). The images were then exported to Slidepath Digital Image (Version 4.0, Leica Biosystems, Wetzlar, Germany) for viewing and analysed using the Masson Trichrome algorithm of Leica Slidepath Tissue Image Analysis software (Version 4.0, Leica Biosystems, Wetzlar, Germany), which quantifies areas of positive Masson Blue, positive Masson Red, and total tissue reference.

#### 4.6.4. Immunohistochemistry

To analyze the osteogenic potential in the calvarial defect side, immunohistochemistry was performed using an antibody specific for osteocalcin (OCN), as previously described [35]. Briefly, in order to deactivate the endogenous peroxidase activity, the sections were immersed in 3% hydrogen peroxide in 0.01 M PBS for 10 min and were then rinsed several times in PBS. Thereafter, sections were blocked with 10% goat normal antiserum (Vector, Burlingame, CA, USA) for 30 min at room temperature. Thereafter, sections were treated with a primary antibody mouse monoclonal (OCG3) to osteocalcin (Abcam, ab13420) overnight at 4 °C. Next, sections were incubated with the secondary antibody, goat anti-mouse IgG (Dako) for 30 min, followed by streptavidin peroxidase for 30 min at room temperature. The sections were incubated in the dark in a chromogen solution containing diaminobenzidine and 0.1% hydrogen peroxide to reveal the immunoreactivity. Finally, the sections were counterstained with hematoxylin and then mounted.

### 4.7. Statistical Analysis

All of the continuous data were first tested for the normality of distribution by Kolmogorov Smirnov statistics. The median, together with interquartile range (IQR), were reported for continuous variables, given the non-normality of the data. Comparisons among different NP groups were performed by the Kruskal−Wallis test and *p* < 0.05 was considered statistically significant. Pairwise comparisons were carried out if a significant result was found by the Kruskall−Wallis test. All of the analyses were done using R 4.0.5 (https://www.R-project.org/, accessed on 31 October 2021).

## 5. Conclusions

In conclusion, the present study provides the first evidence that the application of neutrophils enhances de novo bone formation. The mechanism by which multiple application of neutrophils enhances new bone formation still remains to be elucidated. Continuous application of neutrophils to the defect site may have effects on various immune cells to secrete more effector molecules and growth factors, which are vital for bone tissue healing and regeneration. Further studies are warranted to obtain better insight into the bone regenerative potential of repetitive neutrophil application as a bone tissue engineering strategy. Although our results support the hypothesis that the repetitive application of neutrophils could promote bone regeneration in defects 8 weeks after application, there is still insufficient evidence to substantiate the long-term effects of this cellular therapy in inducing bone healing and formation. Therefore, future long-term in vivo studies should be conducted to validate the findings of the present study.

## Data Availability

The data presented in this study are available within the article text, tables and figures.

## Supplementary Materials

The following are available online at https://www.mdpi.com/article/10.3390/ijms222313016/s1.
