# Supplementary material for: In Vivo Efficacy of Neutrophil-Mediated Bone Regeneration Using a Rabbit Calvarial Defect Model
Source: Int J Mol Sci. 2021 Dec 1;22(23):13016. doi: 10.3390/ijms222313016 (PMC8657540; doi:10.3390/ijms222313016)
Supplement: Supplementary file 1 [file ijms-22-13016-s001.zip › ijms-1468179-supplementary.pdf]

Table S1

	Median (IQR)				
	SA-NP Control	RA-NP Control	SA-NP	RA-NP	P value
**Bone volume at week 4**	7 (6, 9.5)	14 (13, 14.5)	18 (16, 18)	24 (20.5, 25)	**0.032**
**Bone volume at week 8**	17 (15, 20)	20 (16.5, 21)	26 (21.5, 26.5)	30 (28.5, 33.5)	0.067
**BATA at week 4**	16.55 (13.1, 17.39)	22.36 (20.7, 23.45)	23 (22, 25.43)	32.3 (27.72, 33.33)	**0.041**
**BATA at week 8**	24.15 (22.42, 24.97)	26.86 (25.21, 29.34)	27.77 (25.94, 28.78)	37.82 (37.25, 39.66)	0.066
